# Influence of the length of institutionalization on older adults' postural balance and risk of falls: a transversal study^1^

**DOI:** 10.1590/0104-1169.3515.2463

**Published:** 2014

**Authors:** Wagner Oliveira Batista, Edmundo de Drummond Alves, Flávia Porto, Fabio Dutra Pereira, Rosimere Ferreira Santana, Jonas Lírio Gurgel

**Affiliations:** 2 Doctoral student, Escola de Enfermagem Aurora de Afonso Costa, Universidade Federal Fluminense, Niterói, RJ, Brazil; 3 PhD, Associate Professor, Instituto de Educação Física, Universidade Federal Fluminense, Niterói, RJ, Brazil; 4 PhD, Professor, Universidade do Estado do Rio de Janeiro, Rio de Janeiro, RJ, Brazil; 5 MSc, Professor, Universidade Castelo Branco, Rio de Janeiro, RJ, Brazil; 6 PhD, Adjunct Professor, Escola de Enfermagem, Centro de Ciências Médicas, Universidade Federal Fluminense, Niterói, RJ, Brazil; 7 PhD, Adjunct Professor, Instituto de Educação Física, Universidade Federal Fluminense, Niterói, RJ, Brazil

**Keywords:** Aged, Accidental Falls, Postural Balance, Homes for the Aged

## Abstract

**OBJECTIVE:**

to ascertain the influence of the length of institutionalization on older adults' balance and risk of falls.

**METHOD:**

to evaluate the risk of falls, the Berg Balance Scale and the Timed Get Up and Go test were used; and for measuring postural balance, static stabilometry was used, with acquisition of the elliptical area of 95% and mean velocities on the x and y axes of center of pressure displacement. Parametric and nonparametric measures of association and comparison (α<0.05) were used.

**RESULTS:**

there was no significant correlation between the length of institutionalization and the tests for evaluation of risk of falling, neither was there difference between groups and within subgroups, stratified by length of institutionalization and age. In the stabilometric measurements, there was a negative correlation between the parameters analyzed and the length of institutionalization, and difference between groups and within subgroups.

**CONCLUSION:**

this study's results point to the difficulty of undertaking postural control tasks, showing a leveling below the clinical tests' reference scores. In the stabilometric behavior, one should note the reduction of the parameters as the length of institutionalization increases, contradicting the assumptions. This study's results offer support for the development of a multi-professional model for intervention with the postural control and balance of older adults living in homes for the aged.

## Introduction

As well as the advance in age, aging is characterized by an inexorable functional decline of organs and systems, this being influenced by genetic factors, environmental determinants and lifestyle, which act at different levels of complexity^(^ 1 ^)^. This decline, although understood as a physiological process, has posed an important challenge to public health, in the search for maintenance of older adults' functional autonomy^(^ 2 ^)^ and independence and the preservation of their quality of life (QL).

In this regard, abilities of the nervous system, and the sensory interactions with the motor responses, which are determinants for balance and postural control, present a reduction. As a result of this, for an older adult to fall becomes a probable risk^(^ 3 ^)^, reaching over 30% in non-institutionalized older adults; the majority of these older adults are recurrent fallers, which causes multiple harms to the health of this population^(^ 4 ^-^ 5 ^)^.

The increase in longevity has raised various questions for the management of public policies, among which there is the increase in demand for Homes for the Aged (HA). Living in these institutions, however, can promote social isolation, reduction in mental and physical activities, and a worsening in the older adults' QL. Moving to HAs is strongly associated with a decline in the abilities to undertake activities of daily living (ADLs) and a progressive reduction in opportunities for mobility. Thus, for older adults, some activities which are apparently simple, such as walking, can become risky and difficult to undertake. Consequently, this contributes to the failure to undertake ADLs, inducing a hypokinetic routine for them, which becomes an intervening factor for falls^(^ 5 ^)^.

Based on the above, it is appropriate to acknowledge that length of institutionalization (LI) in HAs influences postural control and, as a consequence, the falls. Relating these variables, however, remains little explored in the literature. 

As a result, evaluating routine tasks which require control of posture and balance is fundamental for diagnoses to be made relating to the older adult population's risk of falls, especially when this is in conditions where there are few motor challenges, such as for residents in Has.

This study will contribute to advancing knowledge regarding this issue, that is to say, to gerontology's body of knowledge, providing support for proposing new strategies for multi-professional intervention in HAs, providing opportunities for improving the QL and health of older adults resident in these institutions and, consequently, preventing incapacitating events such as falls. These are considered one of the most important health issues in this age range. 

This study's objective was to ascertain the influence of LI on the postural balance and risk of falls of older adults resident in HAs, through functional tests of postural balance^(^ 6 ^-^ 7 ^)^ and through static stabilometry^(^ 8 ^)^.

## Material and methods

This is an observational study, with a transversal design, undertaken in accordance with the recommendations found in the STrengthening the Reporting of OBservational studies in Epidemiology (STROBE) statement^(^ 9 ^)^. The data were collected between the months of March and June 2012, in two HAs in the municipality of Três Rios in the state of Rio de Janeiro (RJ), Brazil.

This study met all the ethical principles for research involving human beings, as the institutions and participants involved satisfied the requirements of the National Research Ethics Commission, and was approved by the Ethics Committee for Research Involving Human Beings of the Antônio Pedro University Hospital of the Fluminense Federal University (HUAP/UFF), under N. CAAE 0375.0.258.000-11, in conformance with the National Health Council's Resolution N. 466, of 12^th ^December 2012. 

### Population and sample

This study's subjects were selected based on a census in the two HAs in the municipality of Três Rios/RJ (identified as institution A and institution B). At the time of data collection, 96 older adults lived in the two institutions: 38 of them in institution A and 58 in institution B. Both the HAs have philanthropic funding and are supported by the Municipal Department for the Older Adult.

With non-probabilistic sampling, the subjects were subject to the following eligibility criteria: age equal to or over 60 years old; to have autonomy and independence compatible with undertaking tests without external assistance; and to be resident in an institution where the research was undertaken. Factors which made it impossible for the older adult to participate in the tests proposed for this research were adopted as exclusion criteria, with the following being considered: amputation and/or the use of prostheses in the lower limbs; inability to remain standing and to move without support from walking sticks, crutches or other means of assistance. 

When these criteria were applied, 56 older adults were excluded. Thus, 40 subjects met the eligibility criteria, corresponding to - approximately - 42% of the population, made up of 96 residents in the two HA studied. In addition to this there was a sample loss of four older adults (two bedridden, one returned home, and one died) during the data collection period, with 36 subjects remaining until the end of the research (37.5% of the population).

**Figure 1:**
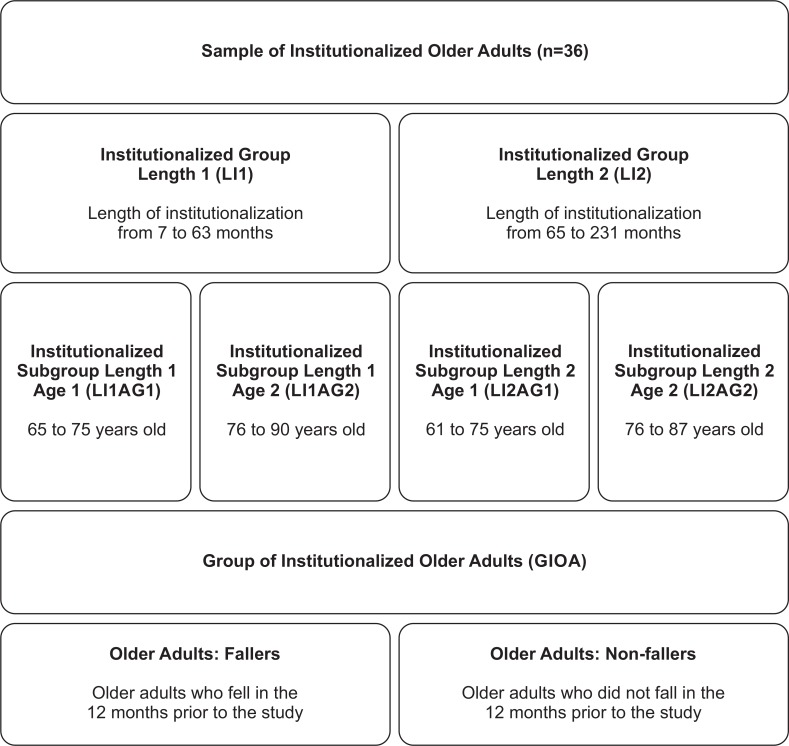
Stratification and homogenization of groups and subgroups of older adults living in homes for the aged. Três Rios, RJ, Brazil, 2012

Using the data obtained from consulting the medical records of the older adults included in the study, the sample was stratified and homogenized by: age ranges, considering the ages between 61 and 90 years old presented in this population; length of institutionalization (LI), whose periods in this sample varied between 07 and 231 months, there being no residents with LI between zero and six months or for exactly 64 months; and falls in the previous year, in which were considered the 12 months which preceded the study for the classification of the older adults as fallers and non-fallers. For these data, the respective medians were taken into consideration, the aim being that the same should have a symmetric division (Figure 1).

### Procedures for evaluating postural balance and risk of falls 

The older adults' risk of falls was evaluated using clinical tests, including the Berg Balance Scale (BBS), in its version translated, adapted and validated for Brazil(6), which consists of a battery of 14 tasks common to the ADLs, which quantitatively evaluate the risk of falls, through observation undertaken by the examiner. For each one of the tasks, a value is attributed, varying between zero and four: zero is applied in the case of the task not being carried out, and four for the best possible performance. The minimum and maximum scores are, respectively, 0 (zero) and 56 points. The higher the score, the lower the risk of falls. 

In the test in question, the tasks are carried out in the following order: seated position to standing; to remain standing without support; to remain seated without support; from standing to seated position; transference from one chair to another; to remain standing up with the eyes shut; to remain standing up with the feet together; standing up, with the arms held forwards; standing up, to pick up an object from the ground; to turn around to look behind; to turn around 360°; position the feet alternatively on a step; to remain standing with one foot forward; and to remain standing on one leg. For this test, the cutoff point of 45 points was adopted, with intraclass correlation coefficients (ICC) for intraexaminer and interexaminer parameters of reliability of 0.99 and 0.98, respectively^(^ 6 ^)^.

The BBS was complemented by the Timed Get up and Go Test (TUG)^(^ 7 ^)^, which is also a test for tracking the older adult population's risk of falls; this has not been transculturally adapted, and is equivalent for measuring because it is a linear metric measurement, in meters (m) and time measurement in seconds (s). This test is widely used, and through it one can measure dynamic balance and walking speed with high intra- and inter-examiner ICC^(^ 10 ^)^. It is carried out in the following way: measurement of the time (s) which an individual takes to get up from the chair, move 3m, go back and turn around to sit in the same chair. In this test, the cutoff point of 20 s was adopted, as recommended by the literature^(^ 3 ^,^ 7 ^,^ 10 ^)^.

For the laboratory evaluation of the balance, static stabilometry was used, which evaluates the postural balance through the quantification of the postural sway in the orthostatic position on a force platform. Normally administered under different protocols, it has a broad application in areas such as rehabilitation, otolaryngology, orthopedics, pharmacology, geriatrics and sport training^(^ 8 ^)^. This test is considered the gold standard in the evaluation of balance and postural control. 

For the test, a force platform was used (0.50m x 0.50m) constructed and calibrated in the Physical Education Institute of the Fluminense Federal University^(^ 11 ^)^. The variables for analysis of the stabilometric test were: displacement and speed of displacement in the medial-lateral (ML) and anterior-posterior (AP) axes from the center of pressure (COP). During the test, the individual adopted the orthostatic posture standing on two feet, barefoot and with the ankles together, forming, between them, an angle of 30°, in line with the markings made on the surface of the force platform, and remained in that position for 45s with the eyes open. In addition to this, the volunteers were requested to keep their eyes fixed on a point established 2.5m from the center of the force platform at the height of their face, such that the individual should keep their head erect (the Frankfurt plane).

For the calculation of the COP, referent to the x (anterior-posterior/AP) and y (medial-lateral/ML) axes, the sample frequency of 100 Hz was adopted. The signals were filtered with a frequency of 50Hz in a Hamming window, using the low-pass FIR filter, so as to reduce possible interferences. The Direct Current (DC) component of the samples was removed and a time cutoff of 30s was made, rejecting the initial and final 7.5 s of the collection; the data were exported to the software MATrix LABoratory (MatLab^(r)^ 7.12.0, R2011a, USA) for the calculation of the elliptical area of 95% of the statokinesiogram (EA/cm^2)^ and stabilogram with the velocity of the AP (Vel x) in cm/s and ML axes (Vel y), in cm/s^(^ 8 ^,^ 12 ^)^.

### Statistical treatment 

Using the Statistical Package for the Social Sciences program 21.0^(r)^ (IBM^(r)^, USA), descriptive statistical analyses (mean, standard deviation, median and interquartile interval) and analyses of distribution (Shapiro-Wilk) were undertaken. The Pearson correlation tests and the Student T test were used for independent samples, and a one-way ANOVA. When necessary, their respective nonparametric tests were applied: the Spearman, Mann-Whitney and Kruskal-Wallis Correlations. For these tests, the level of significance of α<0.05 was adopted.

## Results

The results showed a weak correlation, without significance, between the BBS and TUG tests and LI. The stabilometric parameters, on the other hand, demonstrated significance in the negative correlations between the elliptical area and the speed of the COP in the x axis (Table 1).

**Table 1:** Correlation of the links of institutionalization with balance and the risk of falls in older adults resident in Homes for the Aged. Três Rios, RJ, Brazil, 2012 (N=36)

Variable	Length of institutionalization (month)	p*
Berg Balance Score^†^	-0.264^‡^	0.119
Timed Get up and Go Test**(s)	0.297^§^	0.077
Elliptical Area (cm^2^)	-0.597^‡^	0.003^||^
Mean speed of the COP in the x axis (cm/s)	-0.446^‡^	0.03^||^
Mean speed of the COP in the y axis (cm/s)	-0.279^§^	0.16

* Level of significance

† Score of 0 to 56

‡ Pearson Correlation

§ Spearman Correlation

|| Significant correlation (p=0.05)

When LI1 and LI2 were compared by the measurements of the clinical BBS and TUG tests, these groups did not demonstrate differences. However, when the data from the static stabilometry were compared, a significant difference was perceived in the EA and in the Vel x. In the Vel y, there was no statistical significance and both the velocities showed a reduction in their scales as the LI increased (Table 2).

**Table 2:** Comparison between the LI1 and LI2 groups in balance and risk of falls in residents in Homes for the Aged. Três Rios, RJ, Brazil, 2012

Variable	LI1 (7-63 months)	LI2 (65-231 months)	p
Berg Balance Score*^†^	41.00(17.5)^‡^	38.00(8.8)^‡^	0.261
Timed Get up and Go Test (s)^†^	22.60(15.0)^‡^	21.75(33.1)^‡^	0.763
Elliptical Area (cm^2^)^§^	22.06(10.9)^||^	9.10(7.8)^||^	0.003^¶^
Mean speed of the COP in the x axis (cm/s)^§^	1.99(0.8)^||^	1.32(0.8)^||^	0.032^¶^
Mean speed of the COP in the y axis (cm/s)^†^	2.84(1.36)^‡^	1.47(1.4)^‡^	0.848

* Score of 0 to 56

† Mann-Whitney non-parametric test

‡ Median and interquartile range

§ Student t test

|| Mean and (standard deviation)

¶ Difference between the groups (p=0.05)

The comparisons between the subgroups revealed homoscedasticity in the scores of the BBS and the TUG; when the stabilometric variables were compared, it was possible to see a reduction of their parameters in the direction of the greater LI, as emphasized by the Post hoc test (Table 3).

**Table 3:** Comparison between the subgroups LI1AG1, LI1AG2, LI2AG1 and LI2AG2 of the balance and risk of falls in residents in Homes for the Aged. Três Rios, RJ, Brazil, 2012 (n=9)

Variable	LI1AG1	LI1AG2	LI2AG1	LI2AG2	p	Tukey’s Post hoc test				
						L1AG2	L2AG1		L1AG2	L2AG2
Berg Balance Score*	42.00(15.00)^†^	39.22(18.50)^†^	38.55(9.0)^†^	36.77(4.4)^†^	0.488					
Timed Get up and Go Test**(s)^‡^	24.01(10.7)^†^	25.92(15.3)^†^	26.22(11.86)^†^	32.48(27.1)^†^	0.708					
Elliptical Area (cm^2^)^§^	17.92(11.6)^||^	25.50(10.6)^||^	10.49(3.2)^||^	7.71(4.1)^||^	<0.05^¶^	p=0.05^¶^			p=0.01^¶^	
Mean speed of the COP in the x axis (cm/s)^‡^	1.63(0.9)^†^	2.62(1.7)^†^	1.49(0.7)^†^	1.02(0.7)^†^	>0.05					
Mean speed of the COP in the y axis (cm/s)^‡^	2.64(2.1)^†^	3.11(1.4)^†^	2.14(1.6)^†^	1.41(0.8)^†^	>0.05					

* Score of 0 to 56

† Median and (interquartile range)

‡ Kruskal-Wallis analysis of variance

§ One-way ANOVA

|| Mean and (standard deviation)

¶ Difference between the groups

Significant differences were found in the comparisons undertaken between the older adults who fell and those who did not fall in all the tests suggested (Table 4).

**Table 4:** Comparison between institutionalized older adults who fall and do not fall, resident in Homes for the Aged. Três Rios, RJ, Brazil, 2012

Variable	Faller (n=19) 52.77%	Non-faller (n=17) 47.23%	P
Length of institutionalization (months)	65.00(110.0)*	57.00(59.5)*	0.47^†^
Chronological age	76.18(7.0)^‡^	74.77(7.1)^‡^	0.56^§^
Berg Balance Score^||^	33.00(6.5)^‡^	44.63(6.3)^‡^	0.0001^§¶^
Timed Get up and Go Test**(s)	35.06(16.5)^‡^	20.06(10.1)^‡^	0.002^§¶^
Elliptical Area (cm^2^)	10.71(4.1)^‡^	20.30(7.3)^‡^	0.022^§¶^
Mean speed of the COP in the x axis (cm/s)	1.08(0.8)*	1.88(1.1)*	0.006^†¶^
Mean speed of the COP in the y axis (cm/s)	1.42(1.3)*	2.84(1.4)*	0.016^†¶^

* Median and (interquartile range)

† Mann-Whitney non-parametric test

‡ Mean and (standard deviation)

§ Student t test

|| Score of 0 to 56

¶ Significant difference (P=0.05)

## Discussion

The results demonstrate that the scores for the risk of falls (BBS and TUG) of the population studied (Tables 2, 3 and 4) are below the parameters recommended by the literature in all the groups and subgroups when there is the observance of their cutoff points^(^ 6 ^-^ 7 ^)^; thus demonstrating that these older adults have a considerable probability of suffering falls; as revealed by the prevalence of 52.77% of older adults who fall (Table 4), even with the possibility of underreporting, which may mask an even worse scenario. These results corroborate evidence discovered in other Brazilian cities^(^ 5 ^,^ 13 ^)^, such as was evidenced in a retrospective study^(^ 14 ^)^ which ascertained the prevalence of falls in residents in HAs in São Paulo. In the study, following analysis of 121 hospital records and 87 reports of falls in the period of 12 months, 114 events of falls suffered by 45 older adults were found; with a prevalence of 37.2% of older adults who fall, of whom 46.7% had suffered multiple falls^(^ 14 ^)^.

In the present study, when the results of the clinical tests of evaluation for the risk of falls were associated with the LI, in their respective HAs, weak correlations were found, without statistical significance (Table 1). In the same way, statistically-significant differences were not found in the intragroup (LI1 and LI2) and intra-subgroup (LI1AG1, LI1AG2, LI2AG1 and LI2AG2) comparisons, as shown in Tables 2 and 3. This stratification divided and subdivided the institutionalized older adults by length of residence in the institution and by age. Even though these data did not present statistical significance in the comparisons, it may be perceived that there was a decrease, in nominal terms, of the intragroup and intra-subgroup means (Tables 2 and 3).

These results show a leveling below the scores achieved by the older adults in the clinical tests, in which it is possible to make an inverse analogy to the principle of trainability^(^ 15 ^)^: the more the older adult's capacities are reduced, the more difficult it is to reduce them even further. Another similarity of these physical conditions is reported when one makes a parallel between sedentary aging and the physiological manifestation in the adaptation to the environment of microgravity^(^ 16 ^)^, both introduced by a situation of hypokinesia, leading to a condition of reduced mechanical overload.

The scarcity of correlated works in the literature, using residential time in homes for the aged and/or the chronological age as a manipulated variable, restricts a broader discussion of the results found here. However, when these results are dichotomized for those who fall and those who do not fall, one can note significant differences (Table 4). These findings are in accordance with similar experiments found in the literature^(^ 10 ^,^ 17 ^)^, which - through the BBS and TUG - differentiated the older adults as those who fall or do not fall. The results from the study showed that, in the population studied here, the LI and age are not different between those who fall and those who do not fall, showing a homogeneity of these variables, irrespective of the risk of falls (Table 4).

Although the TUG is used and recognized internationally as an instrument for tracking the risk of falls and its performance is associated with the history of these events, studies indicate that its capacity for predicting this phenomenon remains limited, as does its cutoff point which remains without variations between populations, thus making it a complementary measurement instrument which must be associated with other tests^(^ 10 ^,^ 18 ^)^. In addition to this, the standardization of the test's conditions combined with a control of confounding factors (age, sex and comorbidities) could provide better information regarding the predictive value for falls in older adults^(^ 10 ^,^ 18 ^)^.

In the same way, considering the clinical evaluation tests, the BBS, on its own, cannot definitively predict the risk of a fall, and no cutoff point was identified in its review as an ideal score for the prediction of the risk of a fall. The BBS, therefore, can be considered only as a clinical test which can be used for helping to identify the changes of the risks of falling of elderly patients. This must be used in conjunction with other tests and measures, for a broader evaluation of the risks, in order to guide the safety recommendations and the preventive interventions^(^ 19 ^)^, a condition observed in the present study, in which the above-mentioned test was complemented with the TUG^(^ 7 ^)^ and the static stabilometry^(^ 8 ^)^.

When this study's subjects were subjected to analysis of stabilometric parameters (EA and speed of COP in the x and y axes), the results indicated this study's questions to have greater sensitivity. It was noted that there was a moderate negative correlation with significance between the variables LI and EA, LI and Vel x, and a correlation without significance of LI with Vel y. These results contrast the main evidence of the literature, which indicates that: those who, supposedly, have the greatest postural control sway less, with lower speed of displacement from the COP. Furthermore, older adults and young people differ regarding the geometrical limits of their base of support regarding displacement from the COP^(^ 20 ^)^. There is, however, evidence^(^ 21 ^-^ 23 ^)^ corroborating this study's discoveries, in which the older adults reduce speed and area in order to maintain the efficiency of the task of postural control. This may be related to the fact that, simply, the older adults cannot sway more and with greater speed, so as to be able to maintain a greater margin within the limits of stability.

Another possible explanation for these results is that the cognitive functions may influence the motor task and the risk of falls^(^ 21 ^)^. Authors emphasize that there is an interdependence between postural and cognitive tasks. They suggest that postural control and cognition need common resources and that the inconsistencies in the data and differences in the experimental designs make a broader understanding of the specific mechanisms of posture, cognition and double task difficult. In this scenario, one can include the fear of falling and depression, whose symptoms are a potential predictor of falls in institutionalized older adults^(^ 21 ^-^ 22 ^)^.

The literature warns^(^ 23 ^)^ that the onus of the cerebral abnormalities is correlated significantly with the decline in the control of balance. The signs of the aging of the brain, therefore, are concomitant with the degradation of the cognitive function and the reported age. This fact influences the processing speed of the postural control systems, which influence the integration of information from the sensory motor system in order to produce an action in the appropriate time and with the necessary precision for this function.

In the comparisons established within the groups and within the subgroups (Tables 2 and 3), differences were identified in the stabilometric variables, showing that there is a decrease of these parameters in the direction of those who are older and those with the greatest LI.

Differences were observed in the data from the force platform among older adults who fall and do not fall (p<0.05), in all the stabilometric parameters (Table 4). Emphasis should be placed on the lower elliptical areas and the lower mean speeds of COP of the older adults who suffered one or more falls in the 12 months prior to this study. These results follow the same outcomes as the data discussed previously, which show the tendency for the reduction of the speed and area covered by the COP: which would contradict the studies which divide non-institutionalized older adults between those who fall and those who do not fall by the behavior of the COP, the results of which indicate that those who fall have greater speeds and areas covered by the COP than those who do not fall. Prospective follow-up studies for falls among older adults, which used force platforms, showed there to be a significant correlation of the data with future falls, differentiating the older adults through stabilometry in the: older adults who fall, older adults who do not fall, and those who fall recurrently^(^ 24 ^-^ 25 ^)^. These institutionalized older adults demonstrate the use of different strategies for maintaining postural balance, reducing their sway from the COP in order to dimension their postural control with greater efficiency, suggesting that the motor aspects do not only influence the parameters of postural control, but also the cognitive functions^(^ 23 ^)^.

## Conclusions

In the evaluation of the risk of falls proposed by the BBS and TUG clinical tests, a leveling was observed which was below the minimum established for their respective cutoff points; and, in the stabilometric behavior, a reduction was noticed of the parameters of the COP whenever there is an increase in the LI of the older adults in the HAs, suggesting different strategies for postural control. These discoveries suggest the existence of an association of the LI with postural balance and the risk of falls; difficulty in undertaking motor tasks which require postural control is also indicated. These findings suggest that the length of residence in HAs negatively influences postural balance and, consequently, the risk of falls. This points to the need to rethink health policy for institutionalized older adults, as well as to restructure the methods and strategies used in the care for the health of these individuals. 

Based on the present study's findings, it is recommended that studies be undertaken with other methodological models, which allow the more detailed investigation of the mechanisms which influence these changes. This could contribute to the adoption of a multi-professional model of intervention which is more efficient in the recovery of the health of this specific population. 
